# Modeling Bed Shear Stress Distribution in Rectangular Channels Using the Entropic Parameter

**DOI:** 10.3390/e22010087

**Published:** 2020-01-10

**Authors:** Domenica Mirauda, Maria Grazia Russo

**Affiliations:** 1 School of Engineering, Basilicata University, Viale dell’Ateneo Lucano 10, 85100 Potenza, Italy; 2 Department of Mathematics, Computer Science and Economics, Basilicata University, Viale dell’Ateneo Lucano 10, 85100 Potenza, Italy; mariagrazia.russo@unibas.it

**Keywords:** Tsallis entropy, entropic parameter, bed shear stress distribution, rectangular channels, flow velocity, error analysis

## Abstract

The evaluation of bed shear stress distribution is fundamental to predicting the transport of sediments and pollutants in rivers and to designing successful stable open channels. Such distribution cannot be determined easily as it depends on the velocity field, the shape of the cross section, and the bed roughness conditions. In recent years, information theory has been proven to be reliable for estimating shear stress along the wetted perimeter of open channels. The entropy models require the knowledge of the shear stress maximum and mean values to calculate the Lagrange multipliers, which are necessary to the resolution of the shear stress probability distribution function. This paper proposes a new formulation which stems from the maximization of the Tsallis entropy and simplifies the calculation of the Lagrange coefficients in order to estimate the bed shear stress distribution in open-channel flows. This formulation introduces a relationship between the dimensionless mean shear stress and the entropic parameter which is based on the ratio between the observed mean and maximum velocity of an open-channel cross section. The validity of the derived expression was tested on a large set of literature laboratory measurements in rectangular cross sections having different bed and sidewall roughness conditions as well as various water discharges and flow depths. A detailed error analysis showed good agreement with the experimental data, which allowed linking the small-scale dynamic processes to the large-scale kinematic ones.

## 1. Introduction

Knowledge of the bed shear stress distribution in rivers helps with the evaluation of sediments and pollutants transport, the prediction of erosion and deposition phenomena, the estimation of morphological and geometrical changes, and the calculation of resistance coefficients [1,2,3,4,5,6,7,8,9,10,11]. It can also be useful in planning and designing stable open channels. Such distribution depends on the secondary flows, the shape of the cross section, and the nonuniform roughness around the wetted perimeter. Many researches have focused on the shear stress analysis with experimental, numerical, analytical, and soft computing methods, in channels with different cross-section shapes and both smooth and rough boundaries [12,13,14,15,16,17,18,19,20].

The concept of entropy has been analytically used by various existing methods as a robust theory for estimating the shear stress. However, the entropic approach has been given considerable attention mainly for its ability to evaluate the velocities in open channels [21,22,23,24,25,26,27,28,29,30,31,32], whereas very few studies have been carried out on its application in predicting the shear stress distribution [33,34,35,36,37,38,39,40,41].

Chiu [33] was one of the pioneers who introduced the principle of the maximum entropy and the probability concept to formulate a one-dimensional vertical shear stress distribution law for a steady uniform flow in a wide channel. However, because his study mainly focused on the estimation of velocity profile, the obtained shear stress distribution on the bed was used to evaluate the Lagrange multipliers of the velocity probability density function.

Sterling and Knight [34] attempted to predict the transverse boundary shear stress in circular and trapezoidal channels using Shannon’s entropy [42]. Although the obtained results were in good agreement with the experimental data, their study showed limitations in reflecting the hydraulic behavior of open channels and was unsatisfactory in terms of reliability. This could have been due to the complexity of assigning values to the model parameters, which highly influences the same model output.

Sheikh and Bonakdari [35] proposed a modified Shannon’s entropy application using the power law and a new dimensionless parameter based on the relationship between mean and maximum shear stress. They reported that the simplified equation successfully predicts the shear stress distribution for the investigated cross sections of circular and trapezoidal channels and overcomes the limitations of the previous Shannon’s equation.

In 1961, Renyi [43] introduced the first formal generalization of Shannon’s entropy, which was successively used to study open-channel flows [44,45,46] with similar properties to Shannon’s entropy, for example, it was additive and it had maximum entropy in case of equiprobability. In addition, Renyi’s entropy included an additional parameter which could be used to make it more or less sensitive to the shape of the probability distribution. Khozani and Bonakdari [36] applied a similar theory to derive the shear stress values in circular channels and circular channels with flatbeds and deposited sediments. For circular channels, the model performance was enhanced with increasing flow depth and only deteriorated slightly at the greatest flow depth, while for circular channels with flatbeds it was very good at lower sediment depth.

Tsallis [47] proposed another generalization of Shannon’s entropy which adequately described the statistical features of complex systems in combination with different algorithms. The Tsallis formulation, as well as Renyi’s entropy, contained an additional parameter that provided more flexibility to the shape of the probability distribution function.

Bonakdari et al. [37,38] employed the Tsallis entropy to derive the shear stress distribution in circular and rectangular channels. Their results showed that the Tsallis model is better able to predict the boundary shear stress as compared with the Shannon’s entropy-based model. However, the proposed approach contained two Lagrange parameters that could not be measured directly, involving some inherent degree of statistical uncertainty, which would be dealt with in the work of [39].

The latter evaluated the errors distribution between the experimental shear stresses and the ones obtained from the Tsallis entropy in circular open channels and circular channels with a flatbed for different flow depths by improving the uncertainty procedure proposed by [48] and used by [49]. They introduced the Box–Cox transformation to normalize the shear stresses and four tests (Kolmogorov–Smirnov, Anderson–Darling, Shapiro–Wilk, and Lilliefors) were employed to verify the power of the transformation function in the data normalization. The obtained results showed that the Tsallis model was able to well reproduce the shear stress especially in the circular channels.

Successively, the same authors [40] modified the uncertainty method combining the Johnson transformation function with the Box–Cox transformation in order to analyze the reliability of Shannon’s theory in circular channels with both rigid and alluvial beds. They demonstrated that the prediction of the shear stress in the circular rigid-bed channel is more reliable than the one in the circular alluvial-bed channel.

Recently, Sheikh Khozani and Wan Mohtar [41] employed the Tsallis entropy to estimate the shear stress distribution in a circular channel with a flatbed and in a trapezoidal channel by introducing a new dimensionless parameter as function of the ratio between the mean and maximum shear stress. The model showed a higher performance for predicting shear stress distribution along the wetted perimeter of trapezoidal channels than that of those with a circular shape.

This study proposes a new formulation to evaluate the bed shear stress distribution in rectangular channels. It is based on the following three principles: (1) the maximization of the Tsallis entropy using the Lagrange multipliers, (2) the assumption of a link between the entropic parameter and the Lagrange coefficients of the least-biased probability density function (PDF) of velocity proposed by [50], and (3) the identity between the entropies of the normalized variables which fall in the same range.

In this study, the developed formulation simplifies the calculation of the Lagrange multipliers and introduces a relationship between the dimensionless mean shear stress and the entropic parameter which is based on the ratio between the observed mean and maximum velocity of an open-channel cross section defined by [50] and validated on different open-channel flows. A detailed statistical error analysis was carried out to test the accuracy of the present model. The high correlation with a large set of literature laboratory data showed the good performance of the new approach.

## 2. The Proposed Model

### 2.1. Definition of the Tsallis Entropy and Its Maximization

Tsallis [47] introduced a generalized form of entropy, *H*, for a continuous variable, defined in terms of probability as:

(1) $$H=\frac{1}{q-1}\left[1-\sum_{i=1}^{n}P_{i}^{q}\right]=\frac{1}{q-1}\sum_{i=1}^{n}P_{i}\left(1-P_{i}^{q-1}\right),$$

where *n* is the number of the sampling points, *P_i_* = *P(x_i_)*, *i* = 1, 2, …, *n* are the probabilities of continuous variable *x* = *x_i_*, *i* = 1, 2, …, *n*, and *q* is a real number. *H* assumes the maximum value for *P_i_* = 1/N and for *q* ≥ 0 and the minimum value for *q* < 0. Thus, the dependence of the entropy on the parameter, *q*, can be construed as an indicator of the system complexity. Tsallis showed that, for *q* = 1, Equation (1) converges to Shannon’s entropy. Considering the continuous positive dimensionless shear stress, *τ*/*τ_max_*, Equation (1) becomes:

(2) $$H\left(\frac{\tau }{\tau_{max}}\right)=\frac{1}{q-1}\int_{0}^{1}f\left(\frac{\tau }{\tau_{max}}\right)\left(1-f{\left(\frac{\tau }{\tau_{max}}\right)}^{q-1}\right)d\left(\frac{\tau }{\tau_{max}}\right),$$

where 0 and 1 are the lower and upper bounds of *τ*/*τ_max_*, respectively, and *f*(*τ*/*τ_max_*) is the probability density function. If (1 − *f*(*τ*/*τ_max_*)^q−1^)/(q − 1) is considered as a measure of uncertainty, then Equation (1) or Equation (2) represents the average uncertainty of *τ*/*τ_max_*. The greater the uncertainty, the greater the ignorance, and more information is needed to characterize *τ*/*τ_max_*. In this sense, information and uncertainty regarding dimensionless shear stress *τ*/*τ_max_* are related. Thus, the key in Equation (2) is to derive the least-biased probability density function *f*(*τ*/*τ_max_*) [50,51]. This can be obtained applying the principle of maximum entropy (POME), formulated by Jaynes [52,53,54], to maximize the entropy function subject to specific constraints equal to:

(3) $$\int_{0}^{1}f\left(\frac{\tau }{\tau_{max}}\right)d\left(\frac{\tau }{\tau_{max}}\right)=1,$$

(4) $$\int_{0}^{1}\frac{\tau }{\tau_{max}}f\left(\frac{\tau }{\tau_{max}}\right)d\left(\frac{\tau }{\tau_{max}}\right)=\frac{\tau_{mean}}{\tau_{max}},$$

Equation (4) gives the mean value of *τ*/*τ_max_*. One simple way to achieve the maximization of *H* is using the method of the Lagrange multipliers. To that end, the Lagrange function *L* can be constructed as:

(5) $$L=\int_{0}^{1}\frac{f\left(\frac{\tau }{\tau_{max}}\right)}{q-1}\left(1-f{\left(\frac{\tau }{\tau_{max}}\right)}^{q-1}\right)d\left(\frac{\tau }{\tau_{max}}\right)+\lambda_{1}\left[\int_{0}^{1}f\left(\frac{\tau }{\tau_{max}}\right)d\left(\frac{\tau }{\tau_{max}}\right)-1\right]+\lambda_{2}\left[\int_{0}^{1}\frac{\tau }{\tau_{max}}f\left(\frac{\tau }{\tau_{max}}\right)d\left(\frac{\tau }{\tau_{max}}\right)-\frac{\tau_{mean}}{\tau_{max}}\right],$$

where *λ*_1_ and *λ*_2_ are the Lagrange multipliers. Differentiating Equation (5) according to *f*(*τ*/*τ_max_*), noting f as the variable and *τ*/*τ_max_* as a parameter and equating the derivative to 0, one obtains:

(6) $$\frac{\partial L}{\partial f}=\frac{1}{q-1}[\int_{0}^{1}\left(1-f{\left(\frac{\tau }{\tau_{max}}\right)}^{q-1}\right)d\left(\frac{\tau }{\tau_{max}}\right)+\int_{0}^{1}f\left(\frac{\tau }{\tau_{max}}\right)\left(q+1\right)f{\left(\frac{\tau }{\tau_{max}}\right)}^{q-2}d\left(\frac{\tau }{\tau_{max}}\right)\;+\lambda_{1}\left(\int_{0}^{1}d\left(\frac{\tau }{\tau_{max}}\right)\right)+\lambda_{2}\left(\int_{0}^{1}\frac{\tau }{\tau_{max}}d\left(\frac{\tau }{\tau_{max}}\right)\right)]=0,$$

Neglecting the integration signs, Equation (6) can be written as:

(7) $$\frac{1}{q-1}[\left(1-f{\left(\frac{\tau }{\tau_{max}}\right)}^{q-1}\right)d\left(\frac{\tau }{\tau_{max}}\right)+f\left(\frac{\tau }{\tau_{max}}\right)\left(q+1\right)f{\left(\frac{\tau }{\tau_{max}}\right)}^{q-2}d\left(\frac{\tau }{\tau_{max}}\right)\;+\lambda_{1}d\left(\frac{\tau }{\tau_{max}}\right)+\lambda_{2}\frac{\tau }{\tau_{max}}d\left(\frac{\tau }{\tau_{max}}\right)]=0,$$

Rearranging Equation (7), the probability density function *f(τ*/*τ_max_)* for the dimensionless shear stress is expressed as:

(8) $$f\left(\frac{\tau }{\tau_{max}}\right)={\left[\frac{q-1}{q}\left(\frac{1}{q-1}+\lambda_{1}+\lambda_{2}\frac{\tau }{\tau_{max}}\right)\right]}^{\frac{1}{q-1}},$$

By replacing *λ’* with $\frac{1}{q-1}+\lambda_{1}$, it becomes:

(9) $$f\left(\frac{\tau }{\tau_{max}}\right)={\left[\frac{q-1}{q}\left(\lambda^{\prime }+\lambda_{2}\frac{\tau }{\tau_{max}}\right)\right]}^{\frac{1}{q-1}},$$

Inserting Equation (9) into Equation (2), the entropy function can be rewritten as:

(10) $$H\left(\frac{\tau }{\tau_{max}}\right)=\frac{1}{q-1}\left\{1-\int_{0}^{1}{\left[\frac{q-1}{q}\left(\lambda^{\prime }+\lambda_{2}\frac{\tau }{\tau_{max}}\right)\right]}^{q}d\left(\frac{\tau }{\tau_{max}}\right)\right\}.$$

From Equations (9) and (10), one observes that the probability density function and the entropy function depend on the value of *q* and on the Lagrange multipliers *λ*′ and *λ*_2_. Previous studies of literature [37,50] proposed a feasible range of *q* from 0 to 2. On the basis of the analysis of the shear stress distribution along the wetted perimeter of circular channels and circular channels with flatbed, Bonakdari et al. [37] noted that a larger *q* value leads to more difficulty in solving the equations, and the solutions of the Lagrange parameters for larger *q* values are not as stable as for smaller values. Furthermore, they found that for *q* = 3/4, the parameters λ′ and λ_2_ have simple analytical expressions obtained in a few steps and the results are closer to the experimental ones. Successively, for the same channels Kazemian-Kale-Kale et al. [39] obtained a very good estimation of experimental shear stresses for *q* = 3/4. This value was also applied to predict the shear stress distribution in rectangular [38] and trapezoidal [41] channels. The Lagrange multipliers can be evaluated by substituting Equation (9) into the constraints, Equations (3) and (4):

(11) $${\left[\lambda^{\prime }+\lambda_{2}\right]}^{k}-{\left[\lambda^{\prime }\right]}^{k}=\lambda_{2}k^{k},$$

(12) $$\left(k+1\right)\lambda_{2}{\left[\lambda^{\prime }+\lambda_{2}\right]}^{k}-{\left[\lambda^{\prime }+\lambda_{2}\right]}^{k+1}+{\left[\lambda^{\prime }\right]}^{k+1}=\left(k+1\right)\lambda_{2}^{2}k^{k}\frac{\tau_{mean}}{\tau_{max}},$$

where $k=\frac{q}{q-1}$. Equations (11) and (12) constitute a system of two nonlinear equations in the unknown Lagrange.

### 2.2. Reformulation of the Lagrange Multipliers

Equations (11) and (12) can be solved numerically using observed values of mean and maximum shear stress [37,38,39], which are often difficult to measure directly in open-channel flows. Therefore, this paper proposes a link between the shear stresses and the velocities characterizing their flow, which can be easily acquired. In order to obtain this new formulation, the dimensionless velocity entropy, *H*(*u*/*u_max_*), is introduced, which can be derived analogously as the entropy of the dimensionless shear stress:

(13) $$H\left(\frac{u}{u_{max}}\right)=\frac{1}{m-1}\int_{0}^{1}g\left(\frac{u}{u_{max}}\right)\left(1-g{\left(\frac{u}{u_{max}}\right)}^{m-1}\right)d\left(\frac{u}{u_{max}}\right),$$

where *u_max_* is the maximum velocity of the cross section of an open-channel flow and *g*(*u*/*u_max_*) is equal to:

(14) $$g\left(\frac{u}{u_{max}}\right)={\left[\frac{m-1}{m}\left(a_{1}+a_{2}\frac{u}{u_{max}}\right)\right]}^{\frac{1}{m-1}}.$$

In Equations (13) and (14), *m* is a real number which also varies, like *q*, between zero and two, while *a*_1_ and *a*_2_ are the Lagrange multipliers. In order to simplify the two-dimensional velocity distribution equation Luo and Singh [50], similarly to Chiu in 1988 [55], introduced a new dimensionless entropic parameter, *M_u_*, linked to the Lagrange coefficients as:

(15) $$a_{1}=\frac{4-M_{u}}{2},$$

(16) $$a_{2}=M_{u},$$

obtaining the following expression for the function *H*(*u*/*u_max_*):

(17) $$H\left(\frac{u}{u_{max}}\right)=1-\int_{0}^{1}g\left(\frac{u}{u_{max}}\right)d\left(\frac{u}{u_{max}}\right)=1-\int_{0}^{1}\left[\frac{4-M_{u}}{4}+\frac{M_{u}}{2}\frac{u}{u_{max}}\right]d\left(\frac{u}{u_{max}}\right)=1-\frac{48+M_{u}{}^{2}}{48}=-\frac{M_{u}{}^{2}}{48},$$

The entropic parameter *M_u_* was defined by [50] as function of the ratio between the mean and the maximum velocity of the cross section:

(18) $$M_{u}=12\left(2\frac{u_{mean}}{u_{max}}-1\right).$$

This parameter combines the observed mean and maximum velocities, and thus influences the Lagrange multipliers. It also serves to succinctly express a range of flow characteristics.

Mirauda et al. [56] demonstrated as the entropy of a normalized variable which oscillates between zero and one can be considered formally identical to the entropy of another normalized variable which falls in the same range. Therefore, it is possible to equate the entropy of velocity to the shear stress variable:

(19) $$\frac{1}{q-1}\int_{0}^{1}f\left(\frac{\tau }{\tau_{max}}\right)\left(1-f{\left(\frac{\tau }{\tau_{max}}\right)}^{q-1}\right)d\left(\frac{\tau }{\tau_{max}}\right)=1-\int_{0}^{1}g\left(\frac{u}{u_{max}}\right)d\left(\frac{u}{u_{max}}\right)=-\frac{M_{u}^{2}}{48},$$

As a consequence, the probability density functions that maximize them must be formally the same:

(20) $$f\left(\frac{\tau }{\tau_{max}}\right)=\;\frac{1}{2}\left(a_{1}+a_{2}\frac{\tau }{\tau_{max}}\right),$$

Once the probability density function of *τ*/*τ_max_* has been determined, one can straightforwardly calculate the corresponding mean value:

(21) $$\frac{\tau_{mean}}{\tau_{max}}=\int_{0}^{1}\frac{\tau }{\tau_{max}}f\left(\frac{\tau }{\tau_{max}}\right)d\left(\frac{\tau }{\tau_{max}}\right)=\frac{12+M_{u}}{24},$$

**Figure 1:**
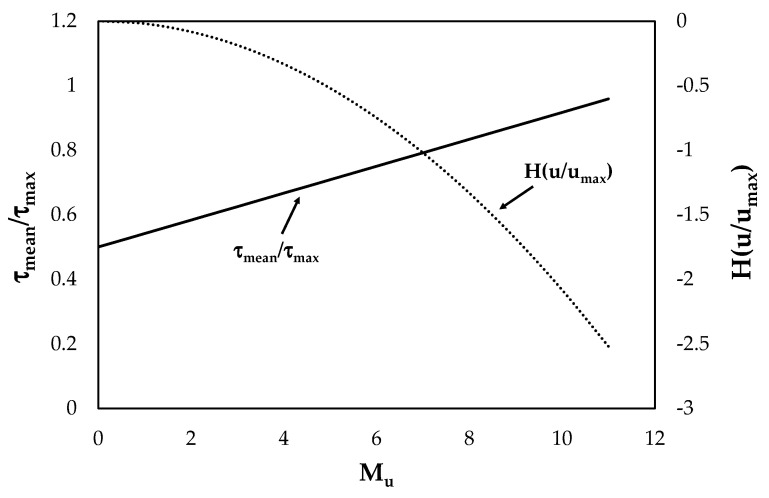
Theoretical trend of the maximum velocity entropy and of the dimensionless mean shear stress for various values of *M_u_*.

In order to evaluate the influence of the parameter *M_u_* on the maximum velocity entropy and dimensionless mean shear stress, *H*(*u*/*u_max_*) and the ratio *τ_mean_*/*τ_max_* are computed from Equations (17) and (21), respectively, for various values of *M_u_*, and plotted in Figure 1. Being the ratio between mean and maximum velocity in the range zero to one, the values of *M_u_* can vary theoretically from −12 to 12 according to Equation (18). Most literature experiences on field and laboratory channels show that the ratio between the mean and maximum velocity in a cross section is always larger than 0.5 and thus *M_u_* can be examined in the interval zero to 12. With these values of the entropic parameter, the theoretical range of *τ_mean_*/*τ_max_*, obtained by Equation (21), is between 0.5 and one, which is almost always observed in open-channel flows. As highlighted in the figure, with increasing entropic parameter, *τ_mean_*/*τ_max_* is inversely proportional to *H*(*u*/*u_max_*). This can be explained by the behavior of various channels both with mobile and fixed bed. In particular, on the one hand, erodible channels generally show an increase of velocity entropies for high values of roughness, width-to-depth ratios, and low slopes [51,56], and a reduction of the shear stresses due to the presence of eroded sediments in the upper part of the flow. On the other hand, artificial channels with rigid boundaries are characterized by low entropies corresponding to significant values of shear stresses because of the lack of suspended load.

Substituting the value of the ratio *τ_mean_*/*τ_max_* in Equation (12), it is possible to obtain a new formulation of the Lagrange multipliers, which estimates the shear stress distribution on the channel bed knowing only one single parameter. It can be expressed as:

(22) $$\left(k+1\right)\lambda_{2}{\left[\lambda^{\prime }+\lambda_{2}\right]}^{k}-{\left[\lambda^{\prime }+\lambda_{2}\right]}^{k+1}+{\left[\lambda^{\prime }\right]}^{k+1}=\frac{1}{2}\left(k+1\right)\lambda_{2}^{2}k^{k}+\frac{M_{u}}{24}\left(k+1\right)\lambda_{2}^{2}k^{k},$$

## 3. Experimental Data

Seven laboratory datasets collected from the literature are adopted in this study to check the accuracy of Equation (21). These include experiences on laboratory rectangular channels characterized by either fixed or mobile bed with varying water discharge and flow depth [57,58,59,60,61,62,63].

Guy et al. [57] collected hydraulic and sediment data in two recirculating flumes, the first one of plywood (2.4 × 0.6 × 46 m) and the second one with clear-plastic sidewalls and a stainless steel floor (0.6 × 0.8 × 18 m), varying the water discharge from zero to 0.623 m^3^/s and from zero to 0.028 m^3^/s, respectively, and adjusting the slope from zero to 1.5% and from zero to 10% accordingly. The investigations were performed using various sand sizes (0.19, 0.27, 0.28, 0.32, 0.33U, 0.33G, 0.45, 0.47, 0.54, and 0.93 mm) coming from rivers, in order to nearly replicate the flow conditions in alluvial channels and phenomena ranging from a plane bed and no sediment movement to violent antidunes. The first step was to make a given discharge of water-sediment mix recirculate at a preselected slope, and after reaching its equilibrium state, then, this was followed by measurements to evaluate the influence of the bed and suspended sediment on the velocity profiles.

Coleman [58] studied the influence of variations in suspended sediment concentrations on velocity profiles in a rectangular Plexiglas channel, 0.356 m wide and 15 m long, adjusting the slope to maintain the fluid discharge (0.064 m^3^/s), average flow depth (0.169 m), and energy gradient constant (0.002). The velocities were initially acquired in conditions of clear water and successively after each sand injection of about 0.91 kg at the upstream end of the flume. The experiments were repeated with three sands of nominal diameters equal to 0.105, 0.210, and 0.420 mm, respectively, and were continued until a highly concentrated and continuously moving sheet of sand was observed on the flume bottom.

Similarly to Coleman’s, Valiani’s point velocities and concentrations were measured in a 0.37 m wide × 10.5 m long laboratory flume for fixed flow rate (0.022 to 0.024 m^3^/s), slope (0.002), and depth (0.10 m) and for changing grain sizes (0.150 mm, 0.106 mm, and 0.075 mm), gradually increasing the solid discharge until saturation of the uniform flow transport capacity [59]. The tests highlighted how the shape of velocity profile is influenced by the presence of sediment suspended.

Lyn [60] analyzed the effect on the flow resistance in a uniform flatbed rectangular flume (13 m long and 0.267 m wide) due to suspended sediment. The point velocity and local wall shear measurements were acquired both in clear water and with suspended sediment, adding well-sorted natural sands of different diameters (d_50_ = 0.15 mm, 0.19 mm, and 0.24 mm). In both saturated (in equilibrium with a sand flatbed) and unsaturated (starved-bed with no sand bed) conditions, Lyn observed how the shape of the velocity-defect profiles was similar to the clear water case, except for a reduction of near-bed velocities compensated either by an increased flow resistance or by increased velocities in the outer region.

Tominaga et al. [61] carried out experiments in a smooth rectangular tilting flume (having a 12.5 m length and 0.4 × 0.4 m cross section) with the bed in a painted iron plate and glass sidewalls. Fully developed (5.07 × 10^4^ ≤ Re ≤ 7.31 × 10^4^), uniform flows were established at the central section of the channel by adjusting the bed slope and the movable weir at the channel end. While the channel width was fixed, the flow depth was changed in order to examine the effect of aspect ratio, *A_r_* (i.e., the ratio between free surface width and flow depth), on secondary currents.

Kironoto and Graf [62] acquired turbulence intensities as well as velocity and Reynolds stress profiles in uniform, turbulent (with Reynolds numbers ranging from 1.6 × 10^5^ to 5.6 × 10^5^), and subcritical (with Froude numbers from 0.23 to 0.48) flows. The experiments were carried out in a tilting channel (16.8 m long, 0.6 m wide, and 0.8 m high), where two different types of roughness were applied on the bed, namely ”rough plate” and ”gravel bed”. The first type of roughness was made up of a single layer of crushed grains with height equal to 0.0048 m glued to the plate fixed on the original steel floor, while the second one was created by a quasi-uniform gravel covering the floor with thickness of about 0.10 m. The low values of the aspect ratio, Ar < 7 (narrow channels), highlighted the influence of the free surface on the sampled sizes.

Graf and Cellino [63] performed experiments in a recirculating tilting channel, 16.8 m long and 0.60 m wide, in conditions of subcritical (0.63 ≤ Fr ≤ 0.85) and turbulent (2.33 × 10^5^ ≤ Re ≤ 3.14 × 10^5^) flows and with values of aspect ratio large enough (A_r_ ≥ 5) to predict a bidimensional flow. The measurements of instantaneous velocity and concentration profiles were made adding sand particles, with diameter of 0.135 mm and 0.230 mm, in several steps to a clear water flow in a uniform condition until the presence of a thin layer of sediments on the bed.

The hydraulic conditions for seven collected experimental datasets are summarized in Table 1.

## 4. Discussion of Results

### 4.1. Validation of Proposed Model

In order to test the validity of the proposed model, a comparison between theoretical shear stress ratios, ${\left(\frac{\tau_{mean}}{\tau_{max}}\right)}_{\left(com\right)}$, and the ones measured, ${\left(\frac{\tau_{mean}}{\tau_{max}}\right)}_{\left(obs\right)}$, for different depths and flow rates in all the investigated laboratory datasets is analyzed. The theoretical shear stress ratios were computed from Equation (21) considering the ranges of the entropic parameter values reported in Table 2.

As observed in the table, the entropic parameter falls into a narrow range of high values and the values of the maximum velocity entropy are low. This could be due to a more uniform velocity distribution and lower aspect ratios in laboratory channels corresponding to higher dimensionless mean shear stresses. Only Guy’s experiences, which are characterized by a greater range of aspect ratio and a behavior more similar to that of rivers, show even lower values of the entropic parameter and higher velocity entropies, as well as a reduction of shear stresses, due to the presence of suspended sediments.

**Figure 2:**
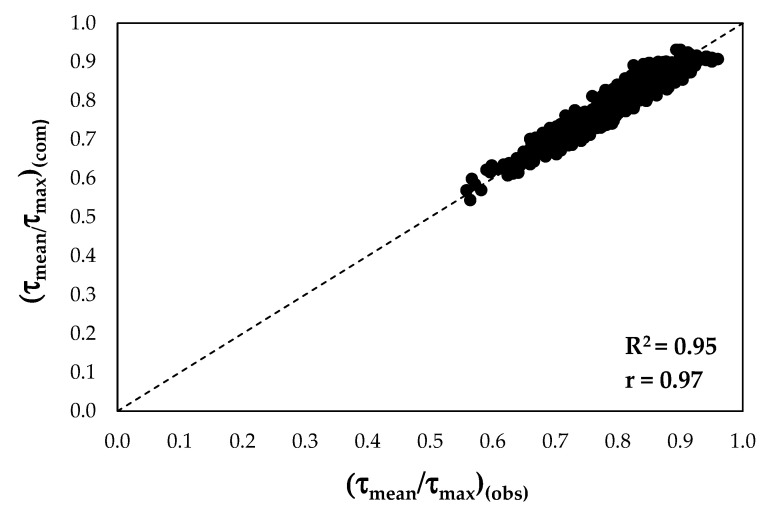
Comparison between the observed values and the predicted ones.

Figure 2 shows the comparison between the theoretical mean dimensionless shear stresses and the observed ones.

As one can see, most of the data are rather close to the 1:1 line with high values of the determination coefficient and of Pearson’s correlation coefficient. Both coefficients underline a perfect positive linear relationship between the simulated and the measured data and less error variance in the observed data explained by the model. To quantify the performance of the model with the collected experimental datasets, different statistical indices such as the root mean square error (RMSE), the RMSE-observations standard deviation ratio (RSR), the mean absolute error (MAE), the percentage of bias (PBIAS), and the Nash–Sutcliffe efficiency (NSE) were selected according to Equations (23)–(27):

(23) $$RMSE=\sqrt{\frac{1}{n}\sum_{i=1}^{n}{\left[\frac{{\left(\frac{\tau_{mean}}{\tau_{max}}\right)}_{\left(com\right)i}-{\left(\frac{\tau_{mean}}{\tau_{max}}\right)}_{\left(obs\right)i}}{{\left(\frac{\tau_{mean}}{\tau_{max}}\right)}_{\left(obs\right)i}}\right]}^{2}},$$

(24) $$RSR=\frac{\sqrt{\sum_{i=1}^{n}{\left[{\left(\frac{\tau_{mean}}{\tau_{max}}\right)}_{\left(com\right)i}-{\left(\frac{\tau_{mean}}{\tau_{max}}\right)}_{\left(obs\right)i}\right]}^{2}}}{\sqrt{\sum_{i=1}^{n}{\left[{\left(\frac{\tau_{mean}}{\tau_{max}}\right)}_{\left(obs\right)i}-{\left(\frac{\tau_{mean}}{\tau_{max}}\right)}_{\left(mean\right)i}\right]}^{2}}},$$

(25) $$MAE=\frac{1}{n}\sum_{i=1}^{n}\left|{\left(\frac{\tau_{mean}}{\tau_{max}}\right)}_{\left(com\right)i}-{\left(\frac{\tau_{mean}}{\tau_{max}}\right)}_{\left(obs\right)i}\right|,$$

(26) $$PBIAS=\left[\frac{\sum_{i=1}^{n}\left[{\left(\frac{\tau_{mean}}{\tau_{max}}\right)}_{\left(obs\right)i}-{\left(\frac{\tau_{mean}}{\tau_{max}}\right)}_{\left(com\right)i}\right]\cdot 100}{\sum_{i=1}^{n}{\left(\frac{\tau_{mean}}{\tau_{max}}\right)}_{\left(obs\right)i}}\right],$$

(27) $$NSE=1-\left[\frac{\sum_{i=1}^{n}{\left[{\left(\frac{\tau_{mean}}{\tau_{max}}\right)}_{\left(obs\right)i}-{\left(\frac{\tau_{mean}}{\tau_{max}}\right)}_{\left(com\right)i}\right]}^{2}}{\sum_{i=1}^{n}{\left[{\left(\frac{\tau_{mean}}{\tau_{max}}\right)}_{\left(obs\right)i}-{\left(\frac{\tau_{mean}}{\tau_{max}}\right)}_{\left(mean\right)i}\right]}^{2}}\right].$$

The root mean square error (RMSE) and the mean absolute error (MAE) are very sensitive to the outliers and they take into account the errors’ distribution. Higher model accuracy generates RMSE and MAE values close to zero. Their advantage is that they show the difference between predicted and observed values in the same units. The RMSE can be substituted at times by its standardized version, RSR, which incorporates the benefits of the same RMSE and includes a scaling/normalization factor such as the standard deviation of the measured data [64]. The percent bias (PBIAS) measures the average tendency of the simulated data to be larger or smaller than the observed ones [65]. The optimal value of PBIAS is 0.0, indicating accurate model simulation, while positive and negative bias values indicate model over- and underestimation, respectively. The Nash–Sutcliffe efficiency (NSE) is a dimensionless technique, which provides the level of model accuracy by determining the relative magnitude of the residual variance as compared with the observed data variance [66]. The NSE shows how well the observed and simulated data fit the 1:1 line. The values of the NSE go from −∞ to 1.0, with NSE = 1 as the optimal value. 0 < NSE < 1 indicates acceptable levels of performance, while NSE ≤0 means unacceptable performance. These last three indices allow assessing the accuracy of the proposed model according to the four categories defined in Table 3 [67].

Table 4 shows how Equation (21) predicts the shear stress ratios for the investigated sections. The values of RSME and MAE highlight the high accuracy of the model, together with the values of RSE, NSE, and PBIAS, which indicate its performance as very good. In addition, the values of PBIAS underline a slight overestimation of the observed data.

### 4.2. Model Performance in Estimating Bed Shear Stress

**Figure 3:**
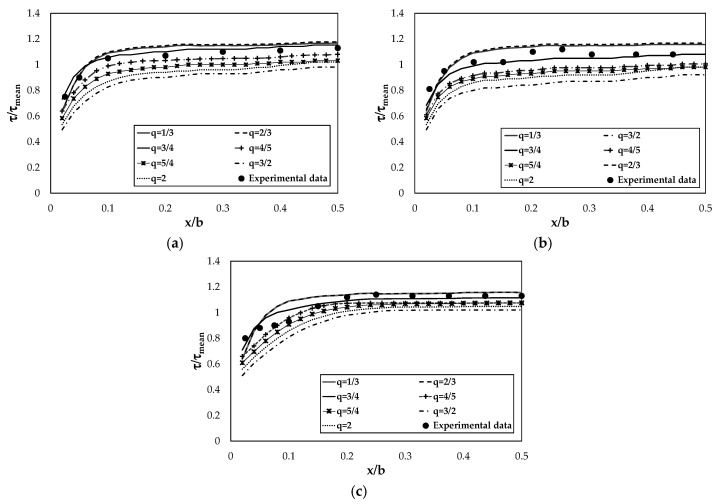
Dimensionless shear stress distribution for various *q* values and different aspect ratios: (**a**) *b*/*h* = 2, (**b**) *b*/*h* = 4, and (**c**) *b*/*h* = 8.

A further validation of the proposed model was carried out for the estimation of the shear stress distribution on the bed of a smooth rectangular cross section using the experimental data of [61]. The new values of the Lagrange coefficients were obtained from Equations (11) and (22), and used to calculate the shear stress distribution on the bed of the cross section through the following expression [38]:

(28) $$\tau =\frac{k}{\lambda_{1}}{\left[{\left(\frac{\lambda^{\prime }}{k}\right)}^{k}+\frac{\lambda_{1}x}{b}\right]}^{1/k}-\frac{\lambda^{\prime }}{\lambda_{1}},$$

where *x* is the path of the channel bed, which changes from zero to *b*/*2*. In this case, shear stress was reported for half of the channel bed considering a symmetric trend on the other half. In order to choose the value of q which can best represent the observed bed shear stresses, their trend was computed for *q* = 1/3, 2/3, 3/4, 4/5, 5/4, 3/2, and 2, as shown in Figure 3. As highlighted in the figure, the shear stress distribution is significantly influenced by the parameter *q*. In fact, for *q* > 3/4 the predicted shear stresses are far from the experimental data, whereas for *q* < 3/4 they have a better agreement. In particular, although *q* = 1/3 and *q* = 2/3 present good results, *q* = 3/4 best fits the observations for all the aspect ratios, as found in the literature. Therefore, the value of *q* is here set to 3/4.

**Figure 4:**
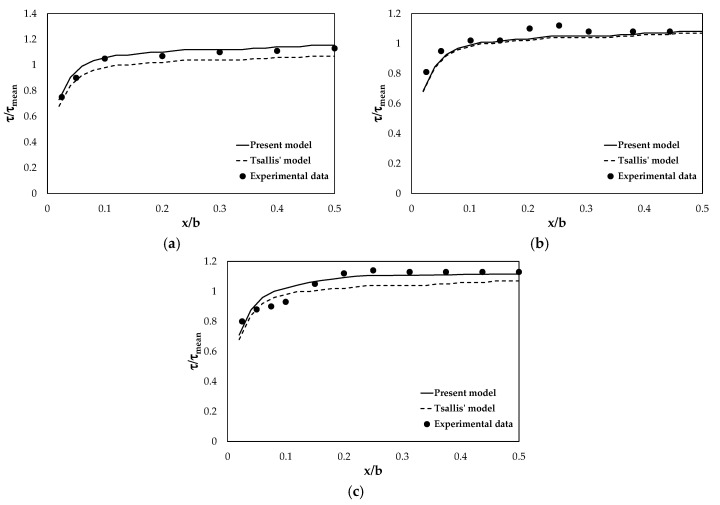
Shear stress distribution on the bed of the cross section for different aspect ratios: (**a**) *b*/*h* = 2, (**b**) *b*/*h* = 4, and (**c**) *b*/*h* = 8.

The results obtained from Equation (28) were compared with those evaluated by applying the classical Tsallis method. Figure 4 shows the shear stress distribution dimensionalized on the mean value against the dimensionless channel width for different aspect ratios, *A_r_*. The straight lines represent the shear stress values estimated by the proposed model, the dashed lines are those deriving from Tsallis method, and the points are the experimental data. As seen in Figure 4, the bed shear stress increases in regions where the secondary current flows towards the wall, and it decreases in regions where the secondary current flows away from the wall [61].

For *A_r_* = 2, the proposed model predictions agree with the laboratory measurements, while the results of the Tsallis method are lower (Figure 4a). In fact, the latter tends to underestimate the bed stresses and this underestimation becomes more significant in the central part of the channel. This highlights how the Tsallis model is more affected by the presence of secondary currents and vortex structures on the channel bed. It is interesting to note that, instead, for *b*/*h* ≈ 4 there is not any difference in the shear stress distribution given by the two models. Both of them show a significant distance from the experimental data in the lateral zone of the channel (Figure 4b). For higher values of the aspect ratio, *A_r_* = 8, the present model displays better performance although it is not able to reproduce the shear stresses near the wall, underlining the influence of the flow conditions changes (Figure 4c). For all values of the aspect ratio, the bed shear stress calculated by the proposed model reaches its maximum value in the center. The Tsallis model indicates slightly lower peak shear stresses. In summary, it is clear from the figures that the proposed model shows a better pattern of the shear stress distribution prediction on the bed of the smooth rectangular cross section.

### 4.3. Simplified Model

Equation (21) can be simplified by introducing, for the same cross section of an open channel, a single value of the entropic parameter, which derives from the best-fit line representing the mean and maximum velocity data measured in different flow conditions. In fact, different studies in the literature have demonstrated that the entropic parameter seems to remain constant over the entire cross section with varying water discharges [6,23,24,68,69] and over the entire reach for rivers with the same morphological characteristics [5]. In particular, Chiu and Said [70] showed that, for a variety of flow rates and depths, a channel cross section seems to have the propensity to establish and maintain an equilibrium state that corresponds to a defined value of the entropic parameter. Therefore, if one considers a single value of the entropic parameter for each investigated section, Equation (21) can be rewritten as:

(29) $$\frac{\tau_{mean}}{\tau_{max}}=\int_{0}^{1}\frac{\tau }{\tau_{max}}f\left(\frac{\tau }{\tau_{max}}\right)d\left(\frac{\tau }{\tau_{max}}\right)=\frac{12+M_{u}^{*}}{24}$$

where $M_{u}^{*}$ is the entropic parameter characteristic for each section.

**Figure 5:**
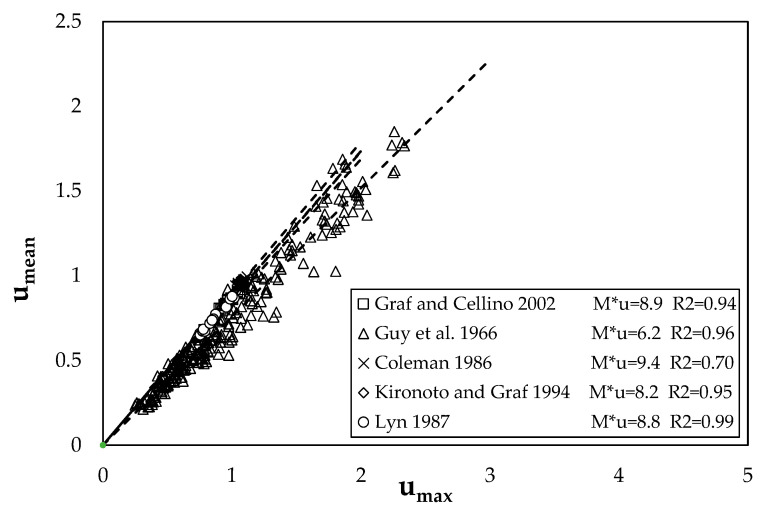
Relationship between the mean and maximum velocity of some investigated cross sections.

Figure 5 shows the dependence between *u_mean_* and *u_ma_*_x_ for some experiments, reported in Table 1. The high value of the determination coefficient, *R*^2^, for all the curves highlights how *M_u_* can be assumed constant for the same cross section with varying water discharge. In fact, substituting in Equation (18) the term *u_mean_*/*u_max_* with the angular coefficient of the best-fit line, one can obtain a single value of the entropic parameter for each investigated section at different flow conditions.

The reliability of Equation (29) was demonstrated by applying the previous statistical error indices, as shown in Table 5.

As seen in Table 5, the simplified model is slightly less accurate although it is categorized by a good to very good performance. The advantage of Equation (29) is that it requires a small amount of input for its resolution, since the evaluation of the shear stress ratios can occur without having to know the point velocities within the cross section with varying water discharge.

## 5. Conclusions

Since the prediction of the shear stress values in open channels is very important to solve a significant number of hydraulic engineering problems, a new formulation, which simplifies the solution of the complex Lagrange multipliers, was developed, in this study, to evaluate the shear stress distribution on the bed of rectangular cross sections. It is based on the following three principles: (1) the maximization of the Tsallis entropy using the Lagrange multipliers, (2) the assumption of a link between the entropic parameter and the Lagrange coefficients of the least-biased probability density function (PDF) of velocity proposed by [50], and (3) the identity between the entropies of the normalized variables which fall in the same range. The application of the new formulation to this shape of the cross section is very interesting since the hydraulic behavior of rivers is often compared to the one of rectangular channels. It proposes a relationship between the dimensionless mean shear stress and the entropic parameter which is based on the ratio between the observed mean and maximum velocity of an open-channel cross section. In addition to simplifying the analytical formulation, the present method also avoids the complicated, time-consuming and expensive measurement of the shear stress in open-channel flows. A large set of literature laboratory data on rectangular cross sections, having different bed and sidewall roughness conditions as well as various water discharges and flow depths, was used to validate the proposed model. The statistical error analysis demonstrated a good agreement between the predicted shear stress ratios and the measured ones, with high values of determination coefficient and Pearson’s correlation coefficient. The indices RSE, NSE, and PBIAS show a very good performance of the model with a slight overestimation of about 9%. In addition, the accuracy of the proposed model was also verified by comparing the dimensionless shear stress distributions on the bed with those estimated through the classical Tsallis method using the experimental data of [61]. According to the results, the present model estimates the bed shear stresses much better than the Tsallis method for all the aspect ratios, which is instead affected more by the presence of the secondary currents and vortex structures on the bottom. Furthermore, a sensitivity analysis allowed choosing the parameter *q* equal to 3/4 as the value able to provide a good evaluation of shear stresses on the channel bed, as already observed in previous literature studies. The high precision of the new model in predicting the shear stress distribution of a rectangular channel shows that it could also be used for other shapes of the cross section. Finally, a further simplification was applied to the present model considering a single value of the entropic parameter for the same cross section with varying water discharge. Despite its minor precision, the simplified model could be used in cross sections of rivers where it is not possible to carry out a detailed and large monitoring activity of the water discharges.

## Figures and Tables

**Table 1:** Geometric, kinematic, and dynamic characteristics of literature data.

*Datasets*	*Q* (*m*^3^*/s*)	*A_r_*	*u_mean_* (*m/s*)	*u_max_* (*m/s*)	*τ_mean_* (*N/m*^2^)	*τ_max_* (*N/m*^2^)
Guy et al. (1966)	0.05–0.64	7.00–29.00	0.21–1.85	0.25–2.34	0.12–14.91	0.15–16.60
Coleman (1986)	0.064	2.04–2.13	0.93–0.99	1.03–1.12	1.60–2.02	2.88–2.98
Valiani (1988)	0.023–0.024	3.63–3.83	0.63–0.66	0.69–0.74	0.69–0.88	0.72–0.96
Lyn (1987)	0.009–0.013	4.05–4.70	0.63–0.87	0.75–1.02	0.88–1.61	0.96–1.88
Tominaga et al. (1989)	0.008–0.015	2.01–8.00	0.19–0.40	0.23–0.46	0.08–0.70	0.11–0.78
Kironoto and Graf (1994)	0.022–0.081	2.07–6.90	0.34–0.50	0.40–0.58	0.21–0.82	0.48–1.53
Graf and Cellino (2002)	0.049–0.065	5.00	0.68–0.92	0.79–1.08	0.25–2.52	0.78–3.02

**Table 2:** Ranges of *u_mean_*/*u_max_* ratios and entropic parameter for all investigated datasets.

*Datasets*	*M_u_*	*H* (*u/u_max_*)	*τ_mean_/τ_max_*
Guy et al. (1966)	1.05–10.19	−0.02–−2.16	0.56–0.93
Coleman (1986)	9.21–9.94	−1.77–−2.06	0.84–0.95
Valiani (1988)	9.45–10.36	−1.86–−2.24	0.89–0.96
Lyn (1987)	8.34–9.17	−1.45–−1.75	0.81–0.92
Tominaga et al. (1989)	8.59–9.48	−1.54–−1.87	0.82–0.92
Kironoto and Graf (1994)	7.51–9.14	−1.17–−1.74	0.78–0.91
Graf and Cellino (2002)	8.23–9.45	−1.41–−1.86	0.82–0.91

**Table 3:** Performance rating of RSR, NSE, and PBIAS.

Performance Rating	RSR	NSE	PBIAS
Very good	0.00 ≤ RSR ≤ 0.50	0.75 < NSE ≤ 1.00	PBIAS < ±10
Good	0.50 < RSR ≤ 0.60	0.65 < NSE ≤ 0.75	±10 ≤ PBIAS < ±15
Satisfactory	0.60 < RSR ≤ 0.70	0.50 < NSE ≤ 0.65	±15 ≤ PBIAS < ±25
Unsatisfactory	RSR > 0.70	NSE ≤ 0.50	PBIAS ≥ ±25

**Table 4:** Statistical indices for the tested proposed model.

Index	Value
**RMSE**	0.32
**MAE**	0.46
**RSR**	0.12
**NSE**	0.93
**PBIAS**	8.65

**Table 5:** Statistical indices for tested simplified model.

Index	Value
**R^2^**	0.92
**r**	0.95
**RMSE**	0.35
**RSR**	0.13
**MAE**	0.60
**NSE**	0.87
**PBIAS**	11.00
